# Quantitative proteomics analysis of *Mycoplasma pneumoniae* identifies potential macrolide resistance determinants

**DOI:** 10.1186/s13568-021-01187-8

**Published:** 2021-02-12

**Authors:** Shaoli Li, Guanhua Xue, Hanqing Zhao, Yanling Feng, Chao Yan, Jinghua Cui, Xianghui Xie, Jing Yuan

**Affiliations:** 1 Department of Bacteriology, Capital Institute of Pediatrics, No. 2 Yabao Road, Chaoyang District, Beijing, 100020 China; 2 Department of Urinary Surgery, Capital Institute of Pediatrics, No. 2 Yabao Road, Chaoyang District, Beijing, 100020 China

**Keywords:** *Mycoplasma pneumoniae*, Drug resistance, Whole proteomes, Quantitative proteomic technique, Differentially expressed proteins

## Abstract

*Mycoplasma pneumoniae* is one of the leading causes of community-acquired pneumonia in children and adolescents. Because of the wide application of macrolides in clinical treatment, macrolide-resistant *M. pneumoniae* strains have become increasingly common worldwide. However, the molecular mechanisms underlying drug resistance in *M. pneumoniae* are poorly understood. In the present work, we analyzed the whole proteomes of macrolide-sensitive and macrolide-resistant strains of *M. pneumoniae* using a tandem mass tag-labeling quantitative proteomic technique, Data are available via ProteomeXchange with identifier PXD022220. In total, 165 differentially expressed proteins were identified, of which 80 were upregulated and 85 were downregulated in the drug-resistant strain compared with the sensitive strain. Functional analysis revealed that these proteins were predominantly involved in protein and peptide biosynthesis processes, the ribosome, and transmembrane transporter activity, which implicates them in the mechanism(s) of resistance of *M. pneumoniae* to macrolides. Our results provide new insights into drug resistance in *M. pneumoniae* and identify potential targets for further studies on resistance mechanisms in this bacterium.

## Key Points

1. Macrolide-resistant *M. pneumoniae* infections are very common worldwide.
2. Quantitative proteomic analysis of macrolide resistance of in *M. pneumoniae.*

## Introduction

*Mycoplasma pneumoniae* causes community-acquired pneumonia in children and adolescents (Saraya 2016). Outbreaks of *M. pneumoniae* infections occur every 3–7 years, and 50–80% of individuals in schools and other semi-enclosed spaces are affected by them. With its ability to survive independently in vitro and with no cell wall, *M. pneumoniae*, a small prokaryotic bacterium, is naturally resistant to drugs that act on cell walls (Waites et al. 2017). Antibiotics that affect the synthesis of bacterial DNA and protein, such as macrolides, quinolones, and tetracycline, can be used to treat *M. pneumoniae* infections. However, tetracycline can cause tooth yellowing, enamel underdevelopment, gastrointestinal tract stimulant reactions, liver toxicity and other side effects, contraindicating its use for children under 8 years of age. Quinolones also cannot be used in children because they can damage cartilage and joints. Therefore, macrolides are currently the first choice treatments for *M. pneumoniae* infections in children (Lee et al. 2018).

Unfortunately, the widespread clinical application of macrolides has triggered microbial resistance to these agents from the 1970s onwards and, since 2000, macrolide-resistant *M. pneumoniae* strains have become increasingly common in many countries, with drug resistance rates reaching 100% in some areas, thereby posing a significant threat to human health (Tanaka et al. 2017; Cao et al. 2017). Previous studies on drug resistance in *M. pneumoniae* have focused on point mutations in the 23S ribosomal gene and L4 and L22 ribosomal proteins, but whether or not changes at the protein level contribute to macrolide resistance awaits investigation (Pereyre et al. 2016; Yang et al. 2017). Here, we used a tandem mass tag (TMT)-labeling-based quantitative proteomic technique to identify differentially expressed proteins (DEPs) in macrolide-sensitive versus macrolide-resistant *M. pneumoniae*.

## Materials and methods

### Chemicals

RIPA Lysis and Extraction Buffer, a TMT10plex Isobaric Label Reagent Set, and a Pierce™ BCA Protein Assay Kit were purchased from Thermo Fisher Scientific. Urea, triethylammonium bicarbonate (TEAB) buffer (1.0 M, pH 8.5 ± 0.1), Tris (2-carboxyethyl) phosphine (TCEP) hydrochloride solution (0.5 M, pH 7.0), iodoacetamide (IAA), formic acid (FA), acetonitrile (ACN), and methanol were purchased from Sigma (St. Louis, MO, USA). Trypsin from bovine pancreas was purchased from Promega (Madison, WI, USA). Ultrapure water was prepared using a Millipore purification system (Billerica, MA, USA).

### Strains

Macrolide-resistant *M. pneumoniae* strain C267 (GenBank No. CP014267), which was isolated in Beijing, China (Li et al. 2016) and the macrolide-sensitive M129 reference strain (ATCC29342) were used in this study. Two strains were cultured in PPLO broth (Becton, Dickinson and company, USA), yeast extract (10%, Oxoid LTD, England), unheated horse serum (20%, Lanzhou national Hyclone Bio-Engineering Co.LTD, China), glucose (50%, CR Double-Crane Pharmaceuticals Co., Ltd, China), phenol red (0.4%, Amresco, OH, USA), and penicillin (1000 U/mL, North China pharmaceutical Group Corporation, China) at 37 °C in a BSL-2 laboratory several days until the color changes. Harvest for protein extraction when the strains (50 mL) had reached logarithmic growth (color changes occurred within 2–3 days after passage).

### Protein extraction and digestion

Proteins were extracted using RIPA Lysis and Extraction Buffer. Protein concentrations were measured using the Pierce BCA Protein Assay Kit. Protein (100 µg) was diluted with 100 mM TEAB to a final volume of 100 µL. TCEP (10 mM) was added to each sample tube and the mixtures were reacted at 56 °C for 1 h. Proteins were alkylated using 20 mM IAA at room temperature in the dark for 1 h. Pre-chilled acetone (− 20 °C, 180 µL) was added and the mixture was stored at − 20 °C overnight. Samples were centrifuged at 8000×*g* for 10 min at 4 °C. The acetone was carefully removed without disturbing the white pellet and the pellet was allowed to dry for 2–3 min. The acetone-precipitated protein pellet (100 µg) was resuspended in 100 µL of 50 mM TEAB. Free trypsin (2 µg) was added to the protein solution and the solution was incubated at 37 °C overnight. Each experiment was repeated three times.

### Labeling and peptide fractionation

Immediately before use, the TMT labeling reagents were equilibrated to room temperature. Anhydrous ACN (41 µL) was added to each tube and the reagent was allowed to dissolve for 5 min with occasional vortexing. The samples were labeled with the TMT reagent. The reaction was incubated for 1 h at room temperature. Hydroxylamine (5%, 8 µL) was added to each sample, and the reactions were quenched over a 15 min period. Samples were combined in equal amounts in fresh microcentrifuge tubes, and the mixed samples were divided into eight fractions using the Pierce™ High pH Reversed-Phase Peptide Fractionation Kit.

### LC–MS/MS analysis and database searching

LC–MS/MS analysis was carried out using a Dionex Ultimate 3000 Nano LC system coupled with a Q-Exactive mass spectrometer (Thermo Fisher Scientific, USA) equipped with an electrospray ionization nanospray source. Mobile phases A and B were 0.1% FA in water and ACN, respectively. The total flow rate was 600 nL/min and a 120-min gradient was set as follows: from 4 to 10% B for 5 min, from 10 to 22% B for 80 min, from 22 to 40% B for 25 min, from 40 to 95% B for 5 min, and held at 95% B for 5 min. The spray voltage was set at 2.0 kV. All MS and MS/MS spectra were acquired in data-dependent acquisition mode and the full mass scan was acquired from *m/z* 300 to 1400 with resolution of 70,000.

The raw MS files (The mass spectrometry proteomics data have been deposited to the ProteomeXchange Consortium via the PRIDE partner repository with the dataset identifier PXD022220 and 10.6019/pxd022220) were analyzed and searched against the UniProt *M. pneumoniae* database using Proteome Discover 2.1 (Thermo Fisher Scientific). Trypsin was selected as the enzyme and up to two missed cleavages were allowed. Cysteine residue alkylation was set as the static modification, and methionine oxidation was set as the variable modification. The mass tolerance of the precursor was 15 ppm and the peptide false discovery rate was controlled at ≤ 1%.

### Bioinformatics analysis

We investigated the Gene Ontology (GO) and Kyoto Encyclopedia of Genes and Genomes (KEGG) pathways of the DEPs using the online OmicsBean resource (http://www.omicsbean.cn/).

### Parallel reaction monitoring analysis

The protein expression levels obtained from the TMT analysis were confirmed by quantifying the expression levels of five selected proteins using Parallel Reaction Monitoring (PRM) analysis. Unique peptides from the target proteins were defined according to the TMT data. The proteins (50 µg) were prepared and digested following the TMT analysis protocol. The obtained peptide mixtures were analyzed by nano LC-PRM MS using easy nano-LC (Thermo Fisher Scientific) coupled to a Q Exactive™ Hybrid Quadrupole-Orbitrap™ Mass Spectrometer (Thermo Fisher Scientific). The raw data were processed using Skyline 2.6, with the cut-off value set to 0.99. The five product ions with the highest signal intensities were allowed to enter each peptide segment for analysis. Each peptide segment was manually integrated, and the results were exported for data analysis.

### Statistical analysis

Statistical analysis was performed using SPSS Statistics software v22.0. Differences in the expression levels of six selected DEPs in the PRM analysis between the macrolide-resistant *M. pneumoniae* strain C267 and the macrolide-sensitive *M. pneumoniae* strain M129 were determined using a *t*-test, and *p* < 0.10 was taken to indicate statistical significance.

## Results

### Identification of DEPs

TMT-labeled quantitative proteomes were determined for *M. pneumoniae* strains C267 and M129. Altogether, 7263 peptides corresponding to 531 proteins were detected. To ensure the reliability of the identification results, we performed peptide length and peptide matching error distribution analyses. Most of the identified peptides were 8–30 amino acids long, and therefore suitable for mass spectrometry (Additional file 1: Figure S1A). The mass error rate for the peptides was ± 20 ppm, which confirmed that the identification was accurate (Additional file 1: Figure S1B).

**Fig. 1:**
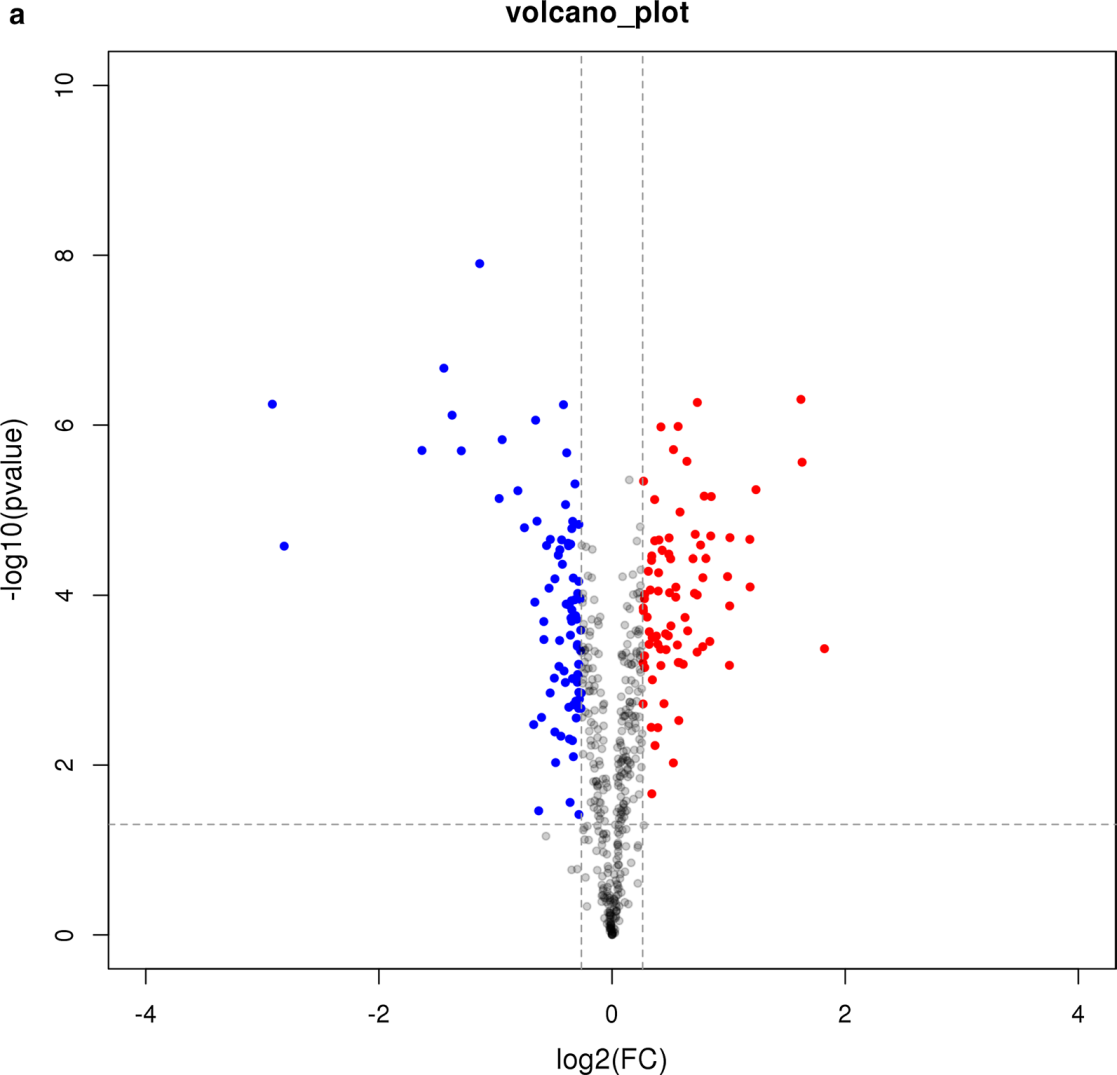

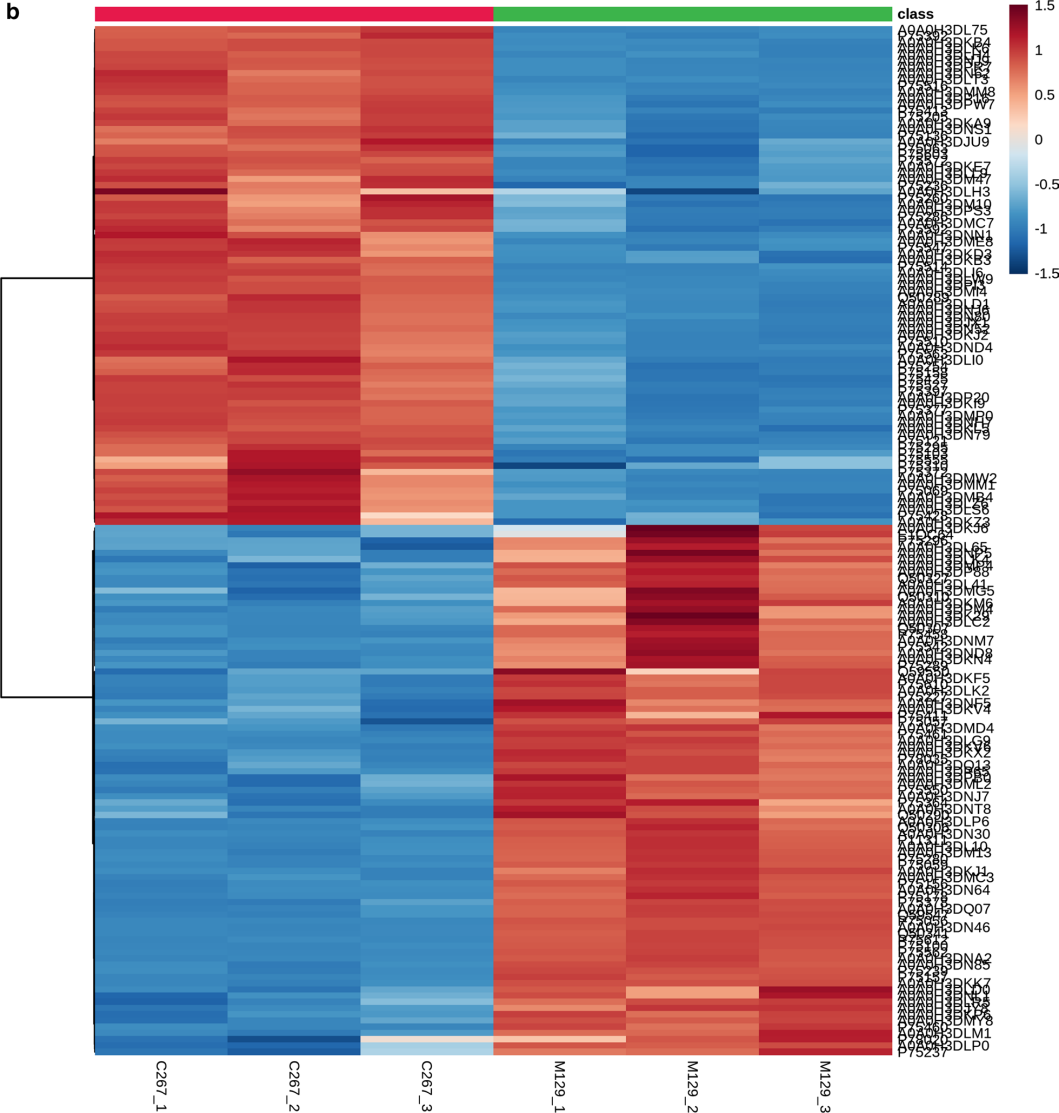
Identification of differentially expressed proteins (DEPs). **a** Volcano plots of the DEPs. The horizontal coordinate indicates the log2(FC) values and the vertical coordinate indicates the −log10 values. **b** Heatmap of DEPs. The expression values shown in different colors denote different protein expression levels

A strict comparison at fold-change ≥ 1.2 (for upregulation) or ≤ 1/1.2 (for downregulation) and a *p*-value cutoff of ≤ 0.05 was applied to identify the DEPs. When comparing macrolide-resistant strain (C267) with macrolide-sensitive strain (M129), 165 DEPs were observed, with 80 upregulated and 85 downregulated (Additional file 2: Table S1). As shown in Fig. 1a and b, a volcano plot and heatmap were employed to analyze the DEPs.

### Functional categorization of DEPs

**Fig. 2:**
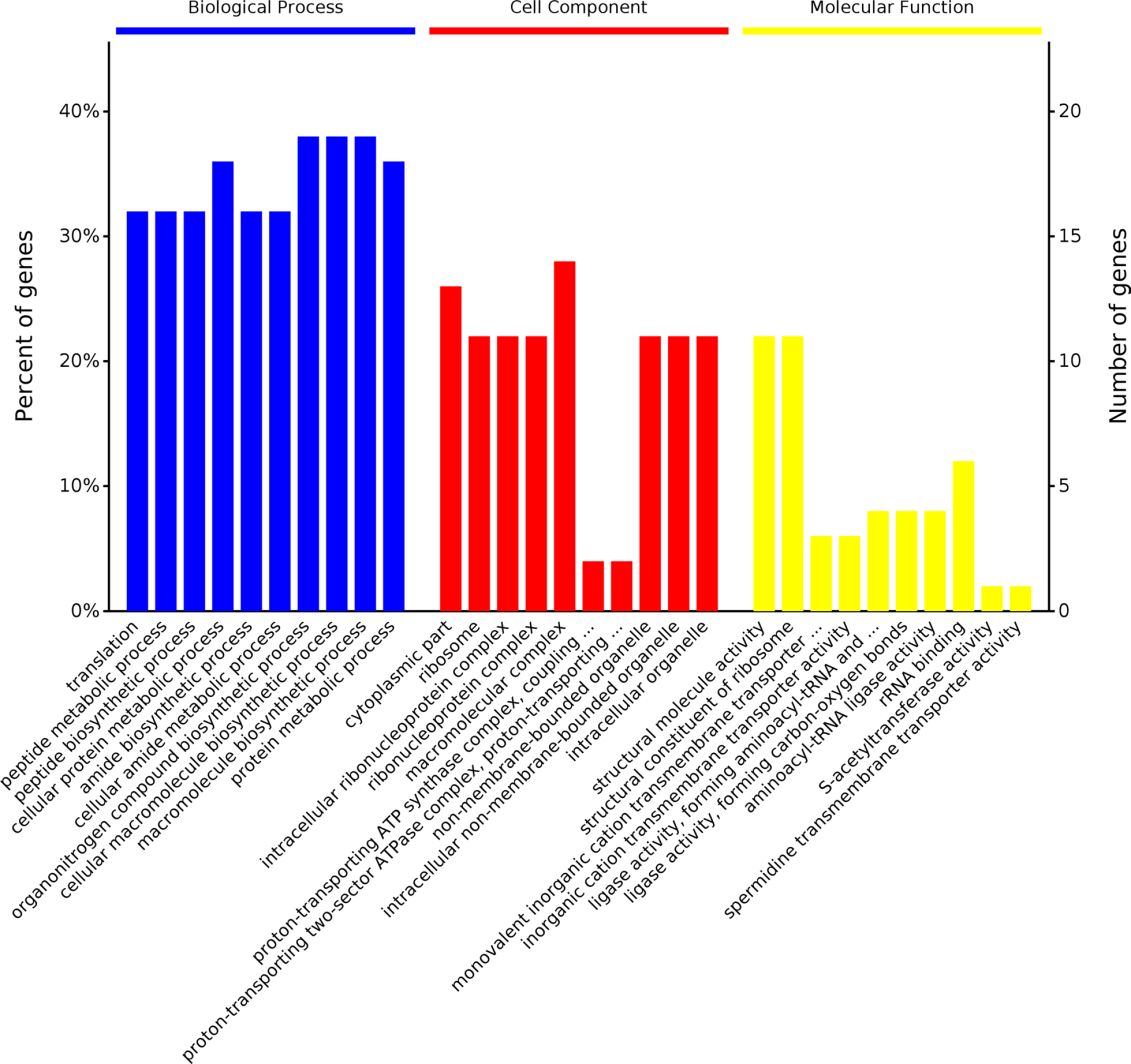
Gene ontology (GO) based on DEPs. Top 10 significant GO terms for the DEPs in each category (sorted by the −log10P-values from high to low). Blue, red and yellow bars represent different GO categories. BP, biological process. CC, cell cellular. MF, molecular function

A broad overview of the main differences between the two strains was obtained in the GO and KEGG analyses. We obtained the top 10 significant GO terms for DEPs in the following categories: biological process (BP), cellular component (CC), and molecular function (MF) (Fig. 2). Translation, cellular protein metabolic process, peptide biosynthetic process, peptide metabolic process, amide biosynthetic process, cellular amide metabolic process, cellular macromolecule biosynthetic process, and macromolecule biosynthetic process were the most significantly enriched in the BP category. In the CC category, cytoplasmic part, ribosome, intracellular ribonucleoprotein complex, ribonucleoprotein complex, and macromolecular complex were found to be significantly enriched. Notably, proton-transporting ATP synthase complex, coupling factor F(o), proton-transporting two-sector ATPase complex, and proton-transporting domain, were among the top 10 significantly enriched CC terms. In the MF category, the following DEPs were highly enriched: structural molecule activity, structural constituent of ribosome, monovalent inorganic cation transmembrane transporter activity, and inorganic cation transmembrane transporter activity terms.

**Fig. 3:**
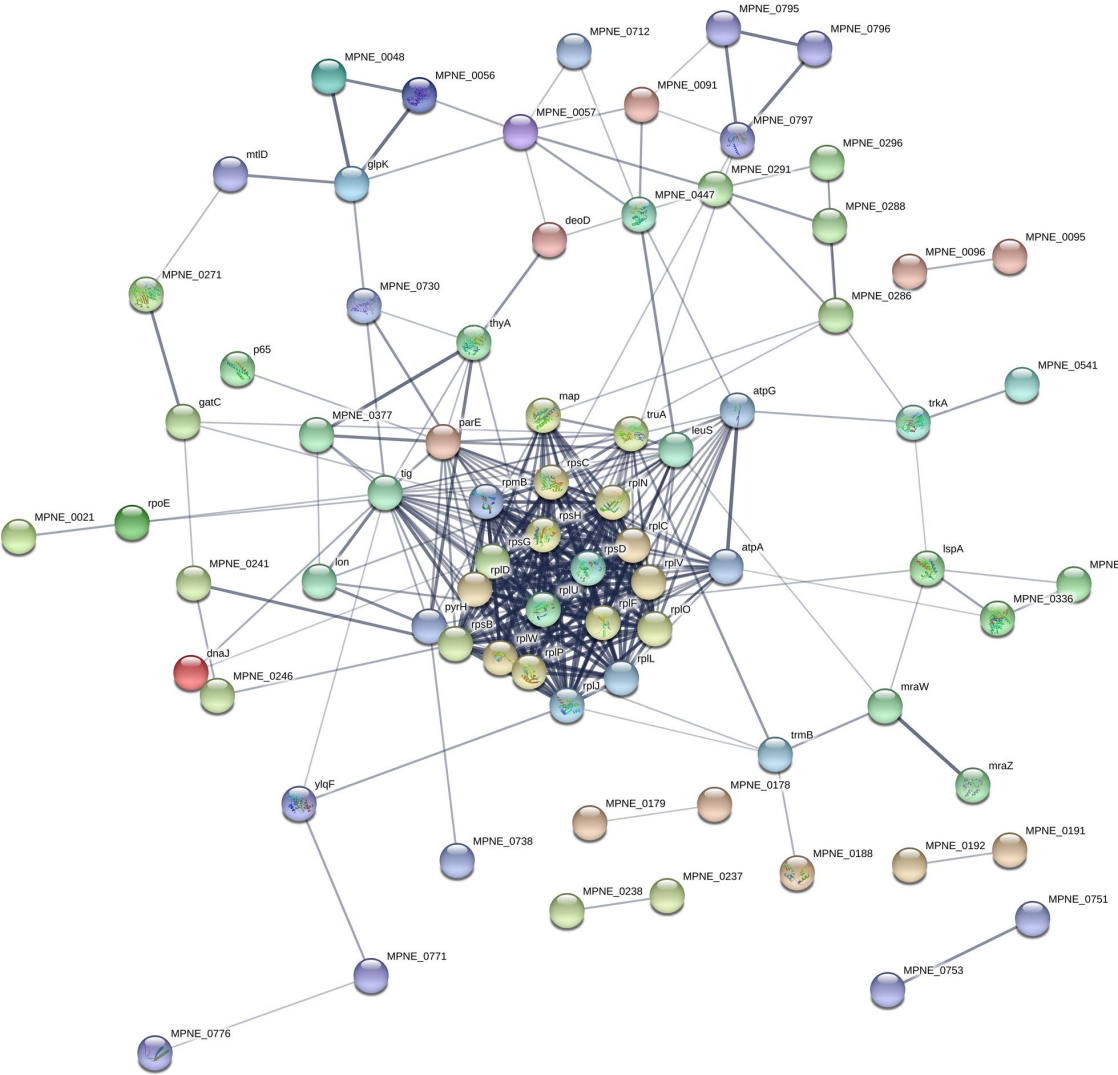
STRING protein network analysis on the proteins identified as being significantly differentiated. Proteins were considered significant at a p-value of < 0.05. The thicker the connecting lines in between the proteins the stronger the protein–protein associations

Mycoplasmas have no cell wall and a small genome; therefore, they lack many enzyme activities found in most bacteria. The absent genes and enzyme function can be supplemented by other genes and their expression products. For these reasons, it is problematic to predict metabolic pathways only by protein annotation, proteome analysis and structural analysis (Pollack 2002). Thirty-two KEGG pathways were annotated for the 165 DEPs (Additional file 3: Table S2). However, it was reported that many metabolic pathways, particularly biosynthetic pathways, are absent in Mycoplasmas, such as those participated in de novo purine biosynthesis and the biosynthesis of amino acids (Pollack et al. 1997; Barile et al. 1966; Himmelreich et al. 1996). They also lack TCA cycle pathway, because they are strict with the nutritional environment, which can be provided by their hosts (Franciele et al. 2013). Table 1 showed the five pathways with the most DEPs. Ribosome pathway was the most significant and abundant pathway. Twenty-eight DEPs were involved with ribosome pathway, including rpsZ, rplW, rpsB, rpsC, rplL, rpsH, rplO, rplU, rplJ, rplK, rplF, rpsD, rplD, rplA, rpmC, rpmB, rplN, rplP, rplV, rplM, rpsT, rplC, rplE, rplX, rpsG, rpmE, rplQ and rpmA. EcfA2, ecfA1, MPN_611, pstA, potC, potA and MPN_058 participated in ABC transporters. A protein interaction network was constructed for the DEPs (Fig. 3). These interactions provide important information about the function and behavior of the DEPs, and are useful to comprehend the resistance mechanisms of *M.pneumniae*.

**Table 1:** KEGG annotation of DEPs between macrolide-resistant strain (C267) and macrolide-sensitive strain (M129)

	Pathway Name	Count	Genes
1	Ribosome	28	rpsZ, rplW, rpsB, rpsC, rplL, rpsH, rplO, rplU, rplJ, rplK, rplF, rpsD, rplD, rplA, rpmC, rpmB, rplN, rplP, rplV, rplM, rpsT, rplC, rplE, rplX, rpsG, rpmE, rplQ, rpmA
2	Metabolic pathways	22	deoD, dnaX, plsY, glpK, tmk, pdhC, csd, ulaE, atpF, nrdE, atpG, MPN_450, rpoE, atpA, pyrH, nrdF, thyA, thiI, ulaF, tpiA, atpE, nadK
3	Pyrimidine metabolism	9	deoD, dnaX, tmk, nrdE, MPN_450, rpoE, pyrH, nrdF, thyA
4	Aminoacyl-tRNA biosynthesis	8	hisS, valS, trpS, glyQS, leuS, pheT, metG, alaS
5	ABC transporters	7	ecfA2, ecfA1, MPN_611, pstA, potC, potA, MPN_058

### Validation of DEPs

Because antibodies suitable for use in *M. pneumoniae* are rare, targeted, quantitative MS approaches such as PRM and multireaction monitoring are essential for DEP confirmation. To validate the results obtained from TMT-based proteomics, we examined the expression levels of several candidate proteins by PRM. Because this technique requires the signature peptide of the target protein to be unique, we selected six proteins with unique signature peptide sequences for PRM analysis. The fold-changes for these proteins differed significantly between the macrolide-resistant C267 strain and the macrolide-sensitive M129 strain at *p* < 0.05, a result in agreement with the findings from the TMT analysis (Table 2).

**Table 2:** Confirmation of the DEPs detected in the TMT analysis using PRM analysis

Accession no.	Gene symbol	Fold-change (C267/M129) in PRM	P-value in PRM	Fold-change (C267/M129) in TMT	P-value in TMT
P75295	MPN_491	12.51738715	1.14223E−05	2.268532831	2.22037E−05
P75121	MPN_670	1.766381125	0.028324373	1.266310644	3.47397E−05
P75603	MPN_090	1.654676801	0.021731425	1.231982961	0.000180854
P75392	pdhC	1.549656015	0.009815993	1.240981292	5.22141E−05
P75527	def	1.514331423	0.036288214	1.302137569	0.000302493
P75236	MPN_542	1.37969945	0.098842902	1.493066835	0.000621553

## Discussion

*Mycoplasma pneumoniae* is one of the main pathogens to cause community-acquired respiratory tract infections and, because these infections can lead to bronchitis and atypical pneumonia as well as a variety of extrapulmonary complications, this pathogen can seriously endanger the health of children and adolescents (Uldum et al. 2012; Principi and Esposito 2013; Waites and Talkington 2004). Because it lacks a cell wall, *M. pneumoniae* is resistant to β-lactams and other antibiotics that act on bacterial cell walls, but it is (in principle) sensitive to macrolides, tetracyclines, and quinolones, because these agents inhibit or affect the synthesis of bacterial proteins and nucleic acids. However, the increasing prevalence of macrolide-resistant *M. pneumoniae* is a significant problem because clinical treatments depend on macrolide antibiotics (Suzuki et al. 2013; Peuchant et al. 2009; Wolff et al. 2008; Dumke et al. 2010; Bajantri et al. 2018). Moreover, some studies have indicated that patients infected with macrolide-resistant strains have greater clinical manifestations and longer disease durations than those infected with wild-type (sensitive) strains (Zhou et al. 2014; Liu et al. 2010). Therefore, research into the drug resistance mechanism(s) of *M. pneumoniae* and implementing rational clinical drug use is now an urgent priority.

**Fig. 4:**
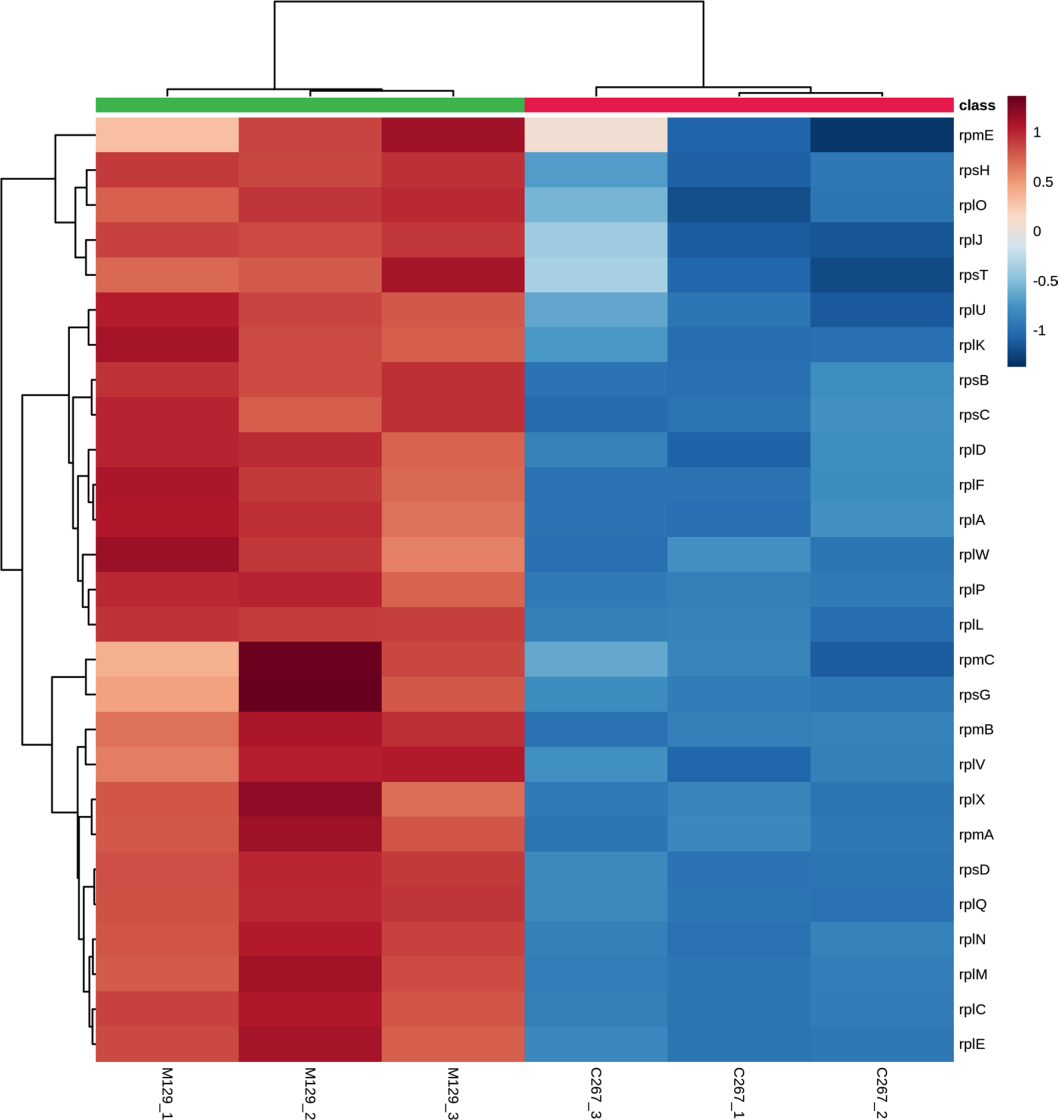
Heatmap of ribosome proteins. The expression values shown in different colors denote the different protein expression levels

Macrolides bind to ribosomal subunits from bacteria and inhibit protein synthesis by blocking peptide transfer and mRNA displacement (Roberts 2004; Giedraitienė et al. 2011). Previous studies on *M. pneumoniae* resistance focused on point mutations in the 23S ribosomal gene and ribosomal proteins L4 and L22 (Principi and Esposito 2013; Matsuoka et al. 2004; Suzuki et al. 2006). Our previous study confirmed that macrolide-resistant strain C267 harbors an A to G mutation at nucleotide position 2063 within domain V of the 23S rRNA gene (Li et al. 2017). In the present study, the GO analysis of DEPs showed that translation, peptide biosynthetic processes, ribosome, intracellular ribonucleoprotein complex, ribonucleoprotein complex, and the structural constituents of ribosomes were significantly enriched terms in the macrolide-resistant strain when compared with the sensitive strain (Fig. 3). The KEGG analysis also revealed that 28 DEPs were involved in ribosomal pathways (Table 1). Notably, all the ribosomal proteins were downregulated in the resistant strain unlike those in the sensitive strain (Fig. 4). Saito et al. (1969) found that erythromycin–ribosome complex formation decreased in erythromycin-resistant *Staphylococcus aureus* strains (Saito et al. 1969). The reduced ability of ribosomes from resistant cells to bind erythromycin and other macrolide antibiotics has been used to demonstrate induced resistance, as has the increased resistance of these ribosomes to inhibition by macrolide antibiotics in cell-free protein synthesis (Shimizu et al. 1970; Weisblum et al. 1971; Allen 1977). Therefore, ribosomal proteins play an important role in drug resistance in *M. pneumoniae*. However, the exact mechanism(s) underpinning the involvement of ribosomal proteins in drug resistance need(s) to be investigated further.

Transporters are another important type of protein involved in drug resistance. Transporters pump a drug out of the cell or the cellular membrane, thereby keeping the intercellular concentrations low (Roberts 2004). An active efflux system, possibly an ABC-type efflux pump, was suggested to be involved in resistance to ciprofloxacin in wall-less *M. hominis* (Raherison et al. 2002). The existence of an active efflux process in *M. hominis* was also implicated in resistance to erythromycin because an ABC transporter inhibitor was able to increase erythromycin uptake levels by more than twofold (Pereyre et al. 2002). Our previous study showed that a macrolide efflux pump, possibly an ABC-type efflux pump, may contribute to macrolide resistance in *M. pneumoniae* C267 (Li et al. 2017). The proteomics results from the present study support this assumption. KEGG analysis of the DEPs between the macrolide-resistant strain C267 and macrolide-sensitive strain M129 indicates that the following seven DEPs are associated with ABC transporters: ECFA2, ECFA1, MPN_611, PSTA, POTC, POTA and MPN_058. In addition, spermidine transmembrane transporter activity, monovalent inorganic cation transmembrane transporter activity, and inorganic cation transmembrane transporter activity were among the 10 most significantly enriched MF terms, suggesting that transporters are significant players in macrolide resistance in *M. pneumoniae*.

Cell membranes consist of a lipid bilayer in which proteins that have important cellular functions, such as receptors, transporters, and enzymes, are embedded (Spector and Yorek 1985). The cell membranes play an important role by acting as a permeability barrier to the entry of diverse chemical agents (Nikaido 2003). Alteration of the cell membrane’s lipid composition can be related to drug resistance. Changed membrane phospholipid and sterol compositions were observed in both clinical and in vitro-adapted azole-resistant *Candida albicans* isolates (Mukhopadhyay et al. 2002; Hitchcock et al. 1986; Kohli et al. 2002; Löffler et al. 2000). It was reported that benzyldimethyltetradecylammonium chloride-adapted *Pseudomonas aeruginosa* cells showed variations in membrane fatty acid composition (Nikaido 2003). In our analysis, two lipid-related pathways, glycerolipid metabolism and glycerophospholipid metabolism, were altered in resistant *M. pneumoniae* C267 compared with sensitive *M. pneumoniae* M129 (Additional file 2: Table S1). Such changes are possibly one of the causes of *M. pneumoniae* drug resistance and, as such, they warrant further investigation.

Drug resistance in *M. pneumoniae* is an increasingly serious problem, and further research into the mechanisms underlying it in this bacterium is urgently needed. Our study provides a global analysis of protein expression changes between the macrolide-resistant C267 strain and the macrolide-sensitive M129 strain of *M. pneumoniae*. We identified several important pathways and candidate proteins that are potential targets for further studies on macrolide resistance in *M. pneumoniae*. However, one limitation of this study is that the sample size was too low. Therefore, our future goal is to test more strains to confirm the relationship between protein expression and drug resistance in *M. pneumoniae*.

## Supplementary Information

**Additional file 1: Figure S1.** Quality control for the mass spectrometry identification process. The peptide length distribution (A) and the mass error distribution (B) are shown.

**Additional file 2: Table S1.** 165 differentially expressed proteins were identified in this study.

**Additional file 3: Table S2.** All KEGG annotation result of DEPs between macrolide-resistantstrain (C267) and macrolide-sensitivestrain (M129).

## Data Availability

The datasets used and/or analyzed during the current study are available from the corresponding author on reasonable request.
