# Supplementary figures and images for: Quantitative proteomics analysis of Mycoplasma pneumoniae identifies potential macrolide resistance determinants
Source: AMB Express. 2021 Feb 12;11:26. doi: 10.1186/s13568-021-01187-8 (PMC7881084; doi:10.1186/s13568-021-01187-8)

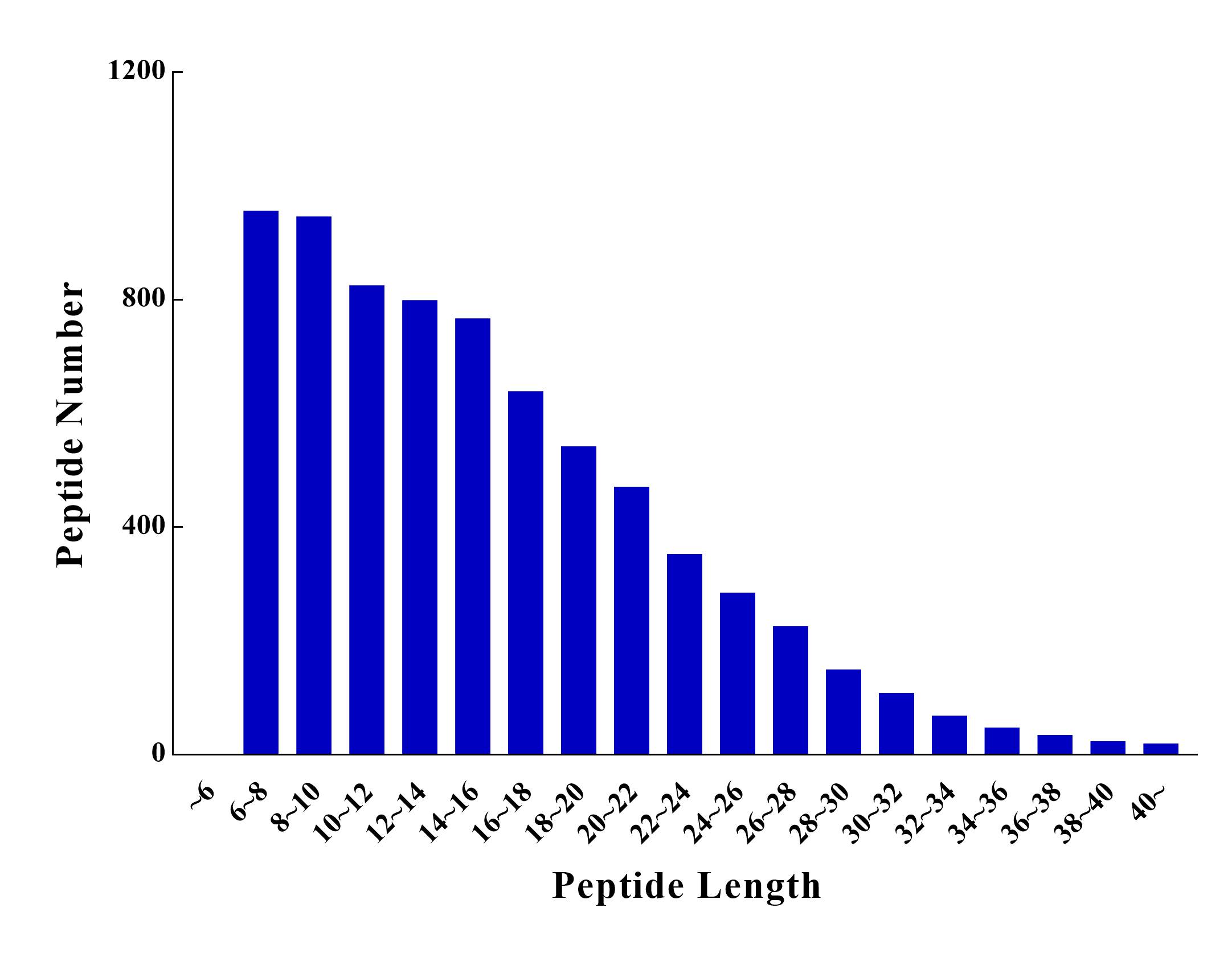

Supplement: Supplementary file 1 — Additional file 1: Figure S1. Quality control for the mass spectrometry identification process. The peptide length distribution (A) and the mass error distribution (B) are shown. [file 13568_2021_1187_MOESM1_ESM.zip › Figuer S1A.png]

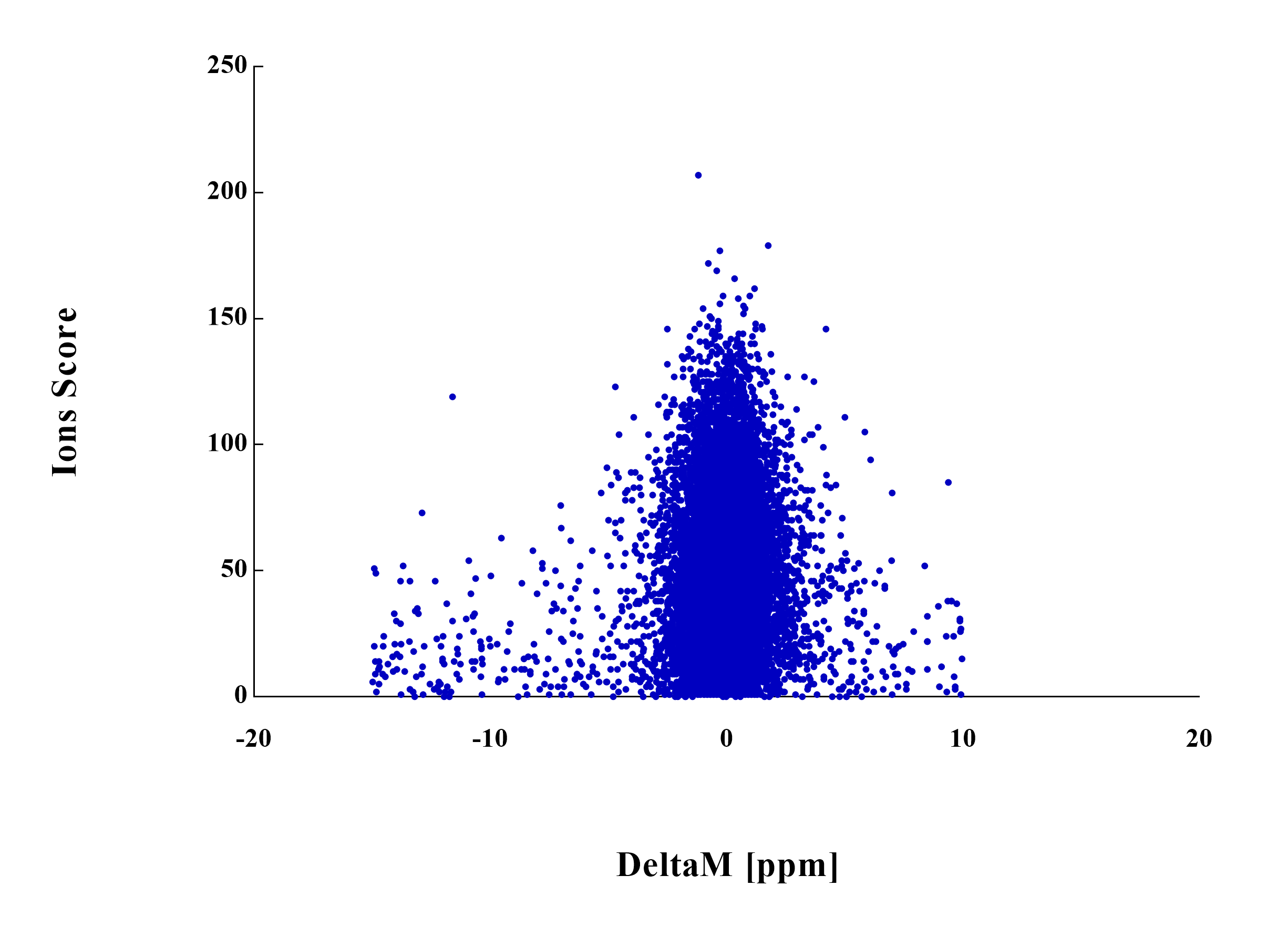

Supplement: Supplementary file 1 — Additional file 1: Figure S1. Quality control for the mass spectrometry identification process. The peptide length distribution (A) and the mass error distribution (B) are shown. [file 13568_2021_1187_MOESM1_ESM.zip › Figure S1B.png]
