# Supplementary material for: Quantitative proteomics analysis of Mycoplasma pneumoniae identifies potential macrolide resistance determinants
Source: AMB Express. 2021 Feb 12;11:26. doi: 10.1186/s13568-021-01187-8 (PMC7881084; doi:10.1186/s13568-021-01187-8)
Supplement: Supplementary file 2 — Additional file 2: Table S1. 165 differentially expressed proteins were identified in this study. [file 13568_2021_1187_MOESM2_ESM.pdf]

AMB Express

Quantitative proteomics analysis of *Mycoplasma pneumoniae* identifies potential macrolide resistance determinants

Shaoli Li[1], Guanhua Xue[1]*, Hanqing Zhao[1], Yanling Feng[1], Chao Yan[1], Jinghua Cui[1], Xianghui Xie[2]*, Jing Yuan[1]*

[1]Department of Bacteriology, Capital Institute of Pediatrics, No. 2 Yabao Road, Chaoyang District, Beijing 100020, China

[2]Department of Urinary Surgery, Capital Institute of Pediatrics, No. 2 Yabao Road, Chaoyang District, Beijing 100020, China

*Corresponding authors: Jing Yuan, E-mail: yuanjing6216@163.com, Telephone: +86-10-85695524; Xianghui Xie, E-mail: xiexianghui@bch.com.cn, Telephone: +86-10-85695126; Guanhua Xue, E-mail: xgh618@163.com, Telephone: +86-10-85695127;

Table S1. 165 differentially expressed proteins were identified in this study.

Name	**Description**	**FC**	**p-value**
A0A0H3DNN1	Uncharacterized protein OS=Mycoplasma pneumoniae (strain ATCC 15531 / DSM 22911 / NBRC 14401 / NCTC 10119 / FH) OX=722438 GN=MPNE_0096 PE=4 SV=1	3.535992022	0.000426135
A0A0H3DPI3	Uncharacterized protein OS=Mycoplasma pneumoniae (strain ATCC 15531 / DSM 22911 / NBRC 14401 / NCTC 10119 / FH) OX=722438 GN=MPNE_0238 PE=4 SV=1	3.095401595	2.73795E-06
A0A0H3DNJ9	Putative D-tyrosyl-tRNA(Tyr) deacylase OS=Mycoplasma pneumoniae (strain ATCC 15531 / DSM 22911 / NBRC 14401 / NCTC 10119 / FH) OX=722438 GN=MPNE_0738 PE=4 SV=1	3.074102032	4.98363E-07
A0A0H3DL75	Uncharacterized protein OS=Mycoplasma pneumoniae (strain ATCC 15531 / DSM 22911 / NBRC 14401 / NCTC 10119 / FH) OX=722438 GN=MPNE_0191 PE=4 SV=1	2.353166839	5.76435E-06
P75397	Uncharacterized protein MG267 homolog OS=Mycoplasma pneumoniae (strain ATCC 29342 / M129) OX=272634 GN=MPN_385 PE=4 SV=1	2.272222775	7.98574E-05
P75295	Uncharacterized protein MPN_491 OS=Mycoplasma pneumoniae (strain ATCC 29342 / M129) OX=272634 GN=MPN_491 PE=4 SV=1	2.268532831	2.22037E-05
A0A0H3DKE7	Uncharacterized protein OS=Mycoplasma pneumoniae (strain ATCC 15531 / DSM 22911 / NBRC 14401 / NCTC 10119 / FH) OX=722438 GN=MPNE_0179 PE=4 SV=1	2.015567069	2.11777E-05
A0A0H3DNS1	Uncharacterized protein OS=Mycoplasma pneumoniae (strain ATCC 15531 / DSM 22911 / NBRC 14401 / NCTC 10119 / FH) OX=722438 GN=MPNE_0527 PE=4 SV=1	2.012374603	0.000133547
A0A0H3DM47	Purine-nucleoside phosphorylase OS=Mycoplasma pneumoniae (strain ATCC 15531 / DSM 22911 / NBRC 14401 / NCTC 10119 / FH) OX=722438 GN=deoD PE=3 SV=1	2.009605748	0.000669299
P75413	UPF0134 protein MPN_368 OS=Mycoplasma pneumoniae (strain ATCC 29342 / M129) OX=272634 GN=MPN_368 PE=3 SV=1	1.98718461	6.0285E-05
A0A0H3DN79	DUF16 domain-containing protein OS=Mycoplasma pneumoniae (strain ATCC 15531 / DSM 22911 / NBRC 14401 / NCTC 10119 / FH) OX=722438 GN=MPNE_0788 PE=4 SV=1	1.803740579	6.94356E-06
P75514	Uncharacterized protein MG121 homolog OS=Mycoplasma pneumoniae (strain ATCC 29342 / M129) OX=272634 GN=MPN_260 PE=4 SV=1	1.798069589	2.02274E-05
A0A0H3DPS3	DUF16 domain-containing protein OS=Mycoplasma pneumoniae (strain ATCC 15531 / DSM 22911 / NBRC	1.788269925	0.000350277

	14401 / NCTC 10119 / FH) OX=722438 GN=MPNE_0398 PE=4 SV=1		
A0A0H3DMP0	Uncharacterized protein OS=Mycoplasma pneumoniae (strain ATCC 15531 / DSM 22911 / NBRC 14401 / NCTC 10119 / FH) OX=722438 GN=MPNE_0188 PE=4 SV=1	1.747099127	3.69611E-05
P75516	Putative carbohydrate transport ATP-binding protein MPN_258 OS=Mycoplasma pneumoniae (strain ATCC 29342 / M129) OX=272634 GN=MPN_258 PE=3 SV=1	1.729458586	6.86991E-06
A0A0H3DLL9	MG032/MG096/MG288 family 2 OS=Mycoplasma pneumoniae (strain ATCC 15531 / DSM 22911 / NBRC 14401 / NCTC 10119 / FH) OX=722438 GN=MPNE_0598 PE=4 SV=1	1.715792026	6.21748E-05
A0A0H3DMC7	DUF16 domain-containing protein OS=Mycoplasma pneumoniae (strain ATCC 15531 / DSM 22911 / NBRC 14401 / NCTC 10119 / FH) OX=722438 GN=MPNE_0565 PE=4 SV=1	1.714734751	0.000403312
P75377	Uncharacterized protein MPN_407 OS=Mycoplasma pneumoniae (strain ATCC 29342 / M129) OX=272634 GN=MPN_407 PE=4 SV=1	1.692938014	2.58817E-05
A0A0H3DKB4	Amino acid or sugar ABC transport system, permease protein OS=Mycoplasma pneumoniae (strain ATCC 15531 / DSM 22911 / NBRC 14401 / NCTC 10119 / FH) OX=722438 GN=MPNE_0291 PE=3 SV=1	1.660507377	5.41846E-07
P75572	UPF0134 protein MPN_204 OS=Mycoplasma pneumoniae (strain ATCC 29342 / M129) OX=272634 GN=MPN_204 PE=3 SV=1	1.659053297	9.92648E-05
A0A0H3DME8	Transporter, major intrinsic protein (MIP) family protein OS=Mycoplasma pneumoniae (strain ATCC 15531 / DSM 22911 / NBRC 14401 / NCTC 10119 / FH) OX=722438 GN=MPNE_0048 PE=3 SV=1	1.658321132	0.000467572
A0A0H3DNH7	Uncharacterized protein OS=Mycoplasma pneumoniae (strain ATCC 15531 / DSM 22911 / NBRC 14401 / NCTC 10119 / FH) OX=722438 GN=MPNE_0543 PE=4 SV=1	1.639769865	1.93371E-05
A0A0H3DN62	Uncharacterized protein OS=Mycoplasma pneumoniae (strain ATCC 15531 / DSM 22911 / NBRC 14401 / NCTC 10119 / FH) OX=722438 GN=MPNE_0776 PE=4 SV=1	1.632496668	9.49214E-05
A0A0H3DJX1	ABC transporter, ATP-binding protein OS=Mycoplasma pneumoniae (strain ATCC 15531 / DSM 22911 / NBRC 14401 / NCTC 10119 / FH) OX=722438 GN=MPNE_0795 PE=4 SV=1	1.618017477	3.71699E-05
P75286	UPF0134 protein MPN_501 OS=Mycoplasma pneumoniae (strain ATCC 29342 / M129) OX=272634 GN=MPN_501 PE=3 SV=1	1.567846157	0.000262608
A0A0H3DLW9	Peptidase S7 domain-containing protein OS=Mycoplasma pneumoniae (strain ATCC 15531 / DSM 22911 /	1.560419551	2.67176E-06

	NBRC 14401 / NCTC 10119 / FH) OX=722438 GN=MPNE_0692 PE=4 SV=1		
A0A0H3DP20	Lipoprotein signal peptidase OS=Mycoplasma pneumoniae (strain ATCC 15531 / DSM 22911 / NBRC 14401 / NCTC 10119 / FH) OX=722438 GN=lspA PE=3 SV=1	1.543237654	0.000182699
P75592	UPF0134 protein MPN_100 OS=Mycoplasma pneumoniae (strain ATCC 29342 / M129) OX=272634 GN=MPN_100 PE=3 SV=1	1.526758698	0.000649941
A0A0H3DP16	Ribosomal RNA small subunit methyltransferase H OS=Mycoplasma pneumoniae (strain ATCC 15531 / DSM 22911 / NBRC 14401 / NCTC 10119 / FH) OX=722438 GN=mraW PE=3 SV=1	1.497898029	1.05829E-05
P75236	Uncharacterized protein MG364 homolog OS=Mycoplasma pneumoniae (strain ATCC 29342 / M129) OX=272634 GN=MPN_542 PE=4 SV=1	1.493066835	0.000621553
P75372	Putative MgpC-like protein MPN_414 OS=Mycoplasma pneumoniae (strain ATCC 29342 / M129) OX=272634 GN=MPN_414 PE=5 SV=1	1.487134774	0.002987579
A0A0H3DLI0	DUF16 domain-containing protein OS=Mycoplasma pneumoniae (strain ATCC 15531 / DSM 22911 / NBRC 14401 / NCTC 10119 / FH) OX=722438 GN=MPNE_0328 PE=4 SV=1	1.482663772	0.000619168
A0A0H3DLK6	Trigger factor OS=Mycoplasma pneumoniae (strain ATCC 15531 / DSM 22911 / NBRC 14401 / NCTC 10119 / FH) OX=722438 GN=tig PE=3 SV=1	1.481064035	1.0384E-06
A0A0H3DLZ6	DUF16 domain-containing protein OS=Mycoplasma pneumoniae (strain ATCC 15531 / DSM 22911 / NBRC 14401 / NCTC 10119 / FH) OX=722438 GN=MPNE_0178 PE=4 SV=1	1.474497494	0.000385406
A0A0H3DKI9	DUF16 domain-containing protein OS=Mycoplasma pneumoniae (strain ATCC 15531 / DSM 22911 / NBRC 14401 / NCTC 10119 / FH) OX=722438 GN=MPNE_0161 PE=4 SV=1	1.461433797	8.02968E-05
A0A0H3DKJ2	Transcriptional regulator MraZ OS=Mycoplasma pneumoniae (strain ATCC 15531 / DSM 22911 / NBRC 14401 / NCTC 10119 / FH) OX=722438 GN=mraZ PE=3 SV=1	1.460531149	0.000105268
A0A0H3DMI4	Basic membrane protein OS=Mycoplasma pneumoniae (strain ATCC 15531 / DSM 22911 / NBRC 14401 / NCTC 10119 / FH) OX=722438 GN=MPNE_0057 PE=4 SV=1	1.440637414	1.94565E-06
P75428	Glycerol-3-phosphate acyltransferase OS=Mycoplasma pneumoniae (strain ATCC 29342 / M129) OX=272634 GN=plsY PE=3 SV=1	1.439760279	0.009432361
P75547	Phosphatidylglycerol--prolipoprotein diacylglyceryl transferase OS=Mycoplasma pneumoniae (strain ATCC	1.419040214	0.000229528

	29342 / M129) OX=272634 GN=lgt PE=3 SV=1		
A0A0H3DLD1	Cof-like hydrolase OS=Mycoplasma pneumoniae (strain ATCC 15531 / DSM 22911 / NBRC 14401 / NCTC 10119 / FH) OX=722438 GN=MPNE_0296 PE=4 SV=1	1.417015592	3.72456E-05
P75205	10 kDa chaperonin OS=Mycoplasma pneumoniae (strain ATCC 29342 / M129) OX=272634 GN=groS PE=3 SV=1	1.407635839	9.34128E-05
A0A0H3DLI6	DUF16 domain-containing protein OS=Mycoplasma pneumoniae (strain ATCC 15531 / DSM 22911 / NBRC 14401 / NCTC 10119 / FH) OX=722438 GN=MPNE_0105 PE=4 SV=1	1.403543713	2.13205E-05
A0A0H3DN80	DJ-1/PfpI family protein OS=Mycoplasma pneumoniae (strain ATCC 15531 / DSM 22911 / NBRC 14401 / NCTC 10119 / FH) OX=722438 GN=MPNE_0336 PE=4 SV=1	1.40161323	3.30856E-05
P75103	UPF0134 protein MPN_010 OS=Mycoplasma pneumoniae (strain ATCC 29342 / M129) OX=272634 GN=MPN_010 PE=1 SV=1	1.397266112	0.000300141
P75254	UPF0134 protein MPN_524 OS=Mycoplasma pneumoniae (strain ATCC 29342 / M129) OX=272634 GN=MPN_524 PE=3 SV=1	1.378684588	0.000436964
A0A0H3DMM1	Uncharacterized protein OS=Mycoplasma pneumoniae (strain ATCC 15531 / DSM 22911 / NBRC 14401 / NCTC 10119 / FH) OX=722438 GN=MPNE_0070 PE=4 SV=1	1.374509612	0.000284719
P75260	UPF0134 protein MPN_138 OS=Mycoplasma pneumoniae (strain ATCC 29342 / M129) OX=272634 GN=MPN_138 PE=3 SV=1	1.36162595	0.001892539
A0A0H3DNJ6	Efflux ABC transporter, permease protein OS=Mycoplasma pneumoniae (strain ATCC 15531 / DSM 22911 / NBRC 14401 / NCTC 10119 / FH) OX=722438 GN=MPNE_0796 PE=4 SV=1	1.347019066	2.97299E-05
A0A0H3DPR7	ABC transporter, ATP-binding protein OS=Mycoplasma pneumoniae (strain ATCC 15531 / DSM 22911 / NBRC 14401 / NCTC 10119 / FH) OX=722438 GN=MPNE_0797 PE=4 SV=1	1.337154202	1.05131E-06
A0A0H3DKD3	Bacterial extracellular solute-binding protein OS=Mycoplasma pneumoniae (strain ATCC 15531 / DSM 22911 / NBRC 14401 / NCTC 10119 / FH) OX=722438 GN=MPNE_0712 PE=4 SV=1	1.336872694	0.000672314
P75069	Histidine--tRNA ligase OS=Mycoplasma pneumoniae (strain ATCC 29342 / M129) OX=272634 GN=hisS PE=1 SV=1	1.333769216	0.000430076
A0A0H3DLT3	Uncharacterized protein OS=Mycoplasma pneumoniae (strain ATCC 15531 / DSM 22911 / NBRC 14401 /	1.321775384	2.25594E-05

	NCTC 10119 / FH) OX=722438 GN=MPNE_0307 PE=4 SV=1		
Q50289	Uncharacterized lipoprotein MPN_199 OS=Mycoplasma pneumoniae (strain ATCC 29342 / M129) OX=272634 GN=MPN_199 PE=3 SV=1	1.31865533	5.43171E-05
A0A0H3DKA9	Uncharacterized protein OS=Mycoplasma pneumoniae (strain ATCC 15531 / DSM 22911 / NBRC 14401 / NCTC 10119 / FH) OX=722438 GN=MPNE_0014 PE=4 SV=1	1.316128927	8.93123E-05
P75063	Uncharacterized protein MG039 homolog OS=Mycoplasma pneumoniae (strain ATCC 29342 / M129) OX=272634 GN=MPN_051 PE=1 SV=1	1.313022764	0.000377532
P75555	Uncharacterized protein MG076 homolog OS=Mycoplasma pneumoniae (strain ATCC 29342 / M129) OX=272634 GN=MPN_214 PE=4 SV=1	1.312922911	0.003629871
P75527	Peptide deformylase OS=Mycoplasma pneumoniae (strain ATCC 29342 / M129) OX=272634 GN=def PE=3 SV=1	1.302137569	0.000302493
P75310	Uncharacterized protein MG328 homolog OS=Mycoplasma pneumoniae (strain ATCC 29342 / M129) OX=272634 GN=MPN_474 PE=4 SV=1	1.291145312	0.005885944
A0A0H3DN52	Glycerol kinase OS=Mycoplasma pneumoniae (strain ATCC 15531 / DSM 22911 / NBRC 14401 / NCTC 10119 / FH) OX=722438 GN=glpK PE=3 SV=1	1.2888225	2.30583E-05
A0A0H3DMM8	dNK domain-containing protein OS=Mycoplasma pneumoniae (strain ATCC 15531 / DSM 22911 / NBRC 14401 / NCTC 10119 / FH) OX=722438 GN=MPNE_0447 PE=4 SV=1	1.28823434	7.53672E-06
A0A0H3DMB4	Uncharacterized protein OS=Mycoplasma pneumoniae (strain ATCC 15531 / DSM 22911 / NBRC 14401 / NCTC 10119 / FH) OX=722438 GN=MPNE_0192 PE=4 SV=1	1.270849327	0.000990298
P75136	UPF0134 protein MPN_655 OS=Mycoplasma pneumoniae (strain ATCC 29342 / M129) OX=272634 GN=MPN_655 PE=3 SV=1	1.26958141	0.000315785
A0A0H3DLH3	CytadhesinP1 domain-containing protein OS=Mycoplasma pneumoniae (strain ATCC 15531 / DSM 22911 / NBRC 14401 / NCTC 10119 / FH) OX=722438 GN=MPNE_0541 PE=4 SV=1	1.266695498	0.02180021
P75121	Uncharacterized protein MG456 homolog OS=Mycoplasma pneumoniae (strain ATCC 29342 / M129) OX=272634 GN=MPN_670 PE=4 SV=1	1.266310644	3.47397E-05
A0A0H3DPW7	FAD dependent oxidoreductase OS=Mycoplasma pneumoniae (strain ATCC 15531 / DSM 22911 / NBRC	1.264699875	3.88412E-05

	14401 / NCTC 10119 / FH) OX=722438 GN=MPNE_0056 PE=4 SV=1		
A0A0H3DKZ3	HTH gntR-type domain-containing protein OS=Mycoplasma pneumoniae (strain ATCC 15531 / DSM 22911 / NBRC 14401 / NCTC 10119 / FH) OX=722438 GN=MPNE_0271 PE=4 SV=1	1.262049212	0.003594386
P75510	Tryptophan--tRNA ligase OS=Mycoplasma pneumoniae (strain ATCC 29342 / M129) OX=272634 GN=trpS PE=1 SV=1	1.2552485	8.67099E-05
P75158	Uncharacterized protein MPN_639 OS=Mycoplasma pneumoniae (strain ATCC 29342 / M129) OX=272634 GN=MPN_639 PE=3 SV=3	1.247686402	0.00026886
P75425	Glycine--tRNA ligase OS=Mycoplasma pneumoniae (strain ATCC 29342 / M129) OX=272634 GN=glyQS PE=3 SV=1	1.246867658	0.000378457
P75392	Dihydrolipoyllysine-residue acetyltransferase component of pyruvate dehydrogenase complex OS=Mycoplasma pneumoniae (strain ATCC 29342 / M129) OX=272634 GN=pdhC PE=1 SV=1	1.240981292	5.22141E-05
P75603	Uncharacterized protein MPN_090 OS=Mycoplasma pneumoniae (strain ATCC 29342 / M129) OX=272634 GN=MPN_090 PE=4 SV=1	1.231982961	0.000180854
A0A0H3DKL5	Competence/damage-inducible protein CinA C-terminal domain protein OS=Mycoplasma pneumoniae (strain ATCC 15531 / DSM 22911 / NBRC 14401 / NCTC 10119 / FH) OX=722438 GN=MPNE_0286 PE=3 SV=1	1.214554727	9.78514E-05
A0A0H3DLS6	Aminotransferase, class V OS=Mycoplasma pneumoniae (strain ATCC 15531 / DSM 22911 / NBRC 14401 / NCTC 10119 / FH) OX=722438 GN=MPNE_0567 PE=3 SV=1	1.214478939	0.000709694
A0A0H3DMW2	ABC transporter, ATP-binding protein OS=Mycoplasma pneumoniae (strain ATCC 15531 / DSM 22911 / NBRC 14401 / NCTC 10119 / FH) OX=722438 GN=MPNE_0021 PE=4 SV=1	1.213580617	0.000517792
A0A0H3DND4	TrkA-N domain protein OS=Mycoplasma pneumoniae (strain ATCC 15531 / DSM 22911 / NBRC 14401 / NCTC 10119 / FH) OX=722438 GN=trkA PE=4 SV=1	1.212556516	0.000109928
A0A0H3DLH4	Leucine--tRNA ligase OS=Mycoplasma pneumoniae (strain ATCC 15531 / DSM 22911 / NBRC 14401 / NCTC 10119 / FH) OX=722438 GN=leuS PE=3 SV=1	1.206035455	4.57697E-06
A0A0H3DKB3	FMN-dependent NADH-azoreductase OS=Mycoplasma pneumoniae (strain ATCC 15531 / DSM 22911 / NBRC 14401 / NCTC 10119 / FH) OX=722438 GN=azoR PE=3 SV=1	1.204862149	0.000152666
P75563	Phenylalanine--tRNA ligase beta subunit OS=Mycoplasma pneumoniae (strain ATCC 29342 / M129)	1.203698125	0.000141354

	OX=272634 GN=pheT PE=3 SV=1		
A0A0H3DM10	OsmC-like protein OS=Mycoplasma pneumoniae (strain ATCC 15531 / DSM 22911 / NBRC 14401 / NCTC 10119 / FH) OX=722438 GN=MPNE_0730 PE=4 SV=1	1.20263289	0.001907198
A0A0H3DJU9	DivIVA domain protein OS=Mycoplasma pneumoniae (strain ATCC 15531 / DSM 22911 / NBRC 14401 / NCTC 10119 / FH) OX=722438 GN=MPNE_0339 PE=4 SV=1	1.201791797	0.000627196
A0A0H3DND8	Methionine aminopeptidase OS=Mycoplasma pneumoniae (strain ATCC 15531 / DSM 22911 / NBRC 14401 / NCTC 10119 / FH) OX=722438 GN=map PE=3 SV=1	0.832831696	0.00142094
P75296	Uncharacterized lipoprotein MG338 homolog OS=Mycoplasma pneumoniae (strain ATCC 29342 / M129) OX=272634 GN=MPN_489 PE=3 SV=1	0.831047269	0.002147897
Q50327	ATP synthase subunit b OS=Mycoplasma pneumoniae (strain ATCC 29342 / M129) OX=272634 GN=atpF PE=1 SV=1	0.829816	0.000257948
A0A0H3DNT8	50S ribosomal protein L23 OS=Mycoplasma pneumoniae (strain ATCC 15531 / DSM 22911 / NBRC 14401 / NCTC 10119 / FH) OX=722438 GN=rplW PE=3 SV=1	0.829126049	0.000449148
A0A0H3DKP6	Ribonucleoside-diphosphate reductase OS=Mycoplasma pneumoniae (strain ATCC 15531 / DSM 22911 / NBRC 14401 / NCTC 10119 / FH) OX=722438 GN=MPNE_0377 PE=3 SV=1	0.825812044	0.000111084
A0A0H3DMD4	ATP synthase gamma chain OS=Mycoplasma pneumoniae (strain ATCC 15531 / DSM 22911 / NBRC 14401 / NCTC 10119 / FH) OX=722438 GN=atpG PE=3 SV=1	0.825606587	0.000108095
P75364	Uncharacterized protein MG296 homolog OS=Mycoplasma pneumoniae (strain ATCC 29342 / M129) OX=272634 GN=MPN_423 PE=1 SV=1	0.823664665	0.001673832
A0A0H3DKJ6	Probable DNA-directed RNA polymerase subunit delta OS=Mycoplasma pneumoniae (strain ATCC 15531 / DSM 22911 / NBRC 14401 / NCTC 10119 / FH) OX=722438 GN=rpoE PE=3 SV=1	0.821121192	0.038211826
A0A0H3DLK2	30S ribosomal protein S2 OS=Mycoplasma pneumoniae (strain ATCC 15531 / DSM 22911 / NBRC 14401 / NCTC 10119 / FH) OX=722438 GN=rpsB PE=3 SV=1	0.820957072	1.48045E-05
A0A0H3DL65	Uncharacterized protein OS=Mycoplasma pneumoniae (strain ATCC 15531 / DSM 22911 / NBRC 14401 / NCTC 10119 / FH) OX=722438 GN=MPNE_0510 PE=4 SV=1	0.820666814	0.001391956
A0A0H3DKF5	30S ribosomal protein S3 OS=Mycoplasma pneumoniae (strain ATCC 15531 / DSM 22911 / NBRC 14401 /	0.820304351	6.86432E-05

	NCTC 10119 / FH) OX=722438 GN=rpsC PE=3 SV=1		
Q50290	Uncharacterized adenine-specific methylase MPN_198 OS=Mycoplasma pneumoniae (strain ATCC 29342 / M129) OX=272634 GN=MPN_198 PE=3 SV=1	0.820029845	0.002151684
A0A0H3DNM7	50S ribosomal protein L7/L12 OS=Mycoplasma pneumoniae (strain ATCC 15531 / DSM 22911 / NBRC 14401 / NCTC 10119 / FH) OX=722438 GN=rplL PE=3 SV=1	0.819499161	0.000650571
A0A0H3DMP4	Uncharacterized protein OS=Mycoplasma pneumoniae (strain ATCC 15531 / DSM 22911 / NBRC 14401 / NCTC 10119 / FH) OX=722438 GN=MPNE_0586 PE=4 SV=1	0.817445069	0.000884781
A0A0H3DMY8	30S ribosomal protein S8 OS=Mycoplasma pneumoniae (strain ATCC 15531 / DSM 22911 / NBRC 14401 / NCTC 10119 / FH) OX=722438 GN=rpsH PE=3 SV=1	0.815222164	9.52184E-05
A0A0H3DLR5	50S ribosomal protein L15 OS=Mycoplasma pneumoniae (strain ATCC 15531 / DSM 22911 / NBRC 14401 / NCTC 10119 / FH) OX=722438 GN=rplO PE=3 SV=1	0.815026193	0.000859023
A0A0H3DML2	50S ribosomal protein L21 OS=Mycoplasma pneumoniae (strain ATCC 15531 / DSM 22911 / NBRC 14401 / NCTC 10119 / FH) OX=722438 GN=rplU PE=3 SV=1	0.813890511	0.000380381
A0A0H3DLP0	50S ribosomal protein L10 OS=Mycoplasma pneumoniae (strain ATCC 15531 / DSM 22911 / NBRC 14401 / NCTC 10119 / FH) OX=722438 GN=rplJ PE=3 SV=1	0.813617765	0.001964627
A0A0H3DNF5	tRNA (guanine-N(7)-)-methyltransferase OS=Mycoplasma pneumoniae (strain ATCC 15531 / DSM 22911 / NBRC 14401 / NCTC 10119 / FH) OX=722438 GN=trmB PE=3 SV=1	0.813404042	0.001058905
A0A0H3DKN4	Ribosome biogenesis GTPase A OS=Mycoplasma pneumoniae (strain ATCC 15531 / DSM 22911 / NBRC 14401 / NCTC 10119 / FH) OX=722438 GN=ylqF PE=3 SV=1	0.813278317	0.000398134
P75550	50S ribosomal protein L11 OS=Mycoplasma pneumoniae (strain ATCC 29342 / M129) OX=272634 GN=rplK PE=3 SV=3	0.810483949	0.000191826
P75411	Putative adhesin P1-like protein MPN_370 OS=Mycoplasma pneumoniae (strain ATCC 29342 / M129) OX=272634 GN=MPN_370 PE=5 SV=1	0.807991746	0.002792172
A0A0H3DLD0	E1-E2 ATPase OS=Mycoplasma pneumoniae (strain ATCC 15531 / DSM 22911 / NBRC 14401 / NCTC 10119 / FH) OX=722438 GN=MPNE_0241 PE=4 SV=1	0.80639892	0.001763765
A0A0H3DL41	DnaJ domain protein OS=Mycoplasma pneumoniae (strain ATCC 15531 / DSM 22911 / NBRC 14401 / NCTC	0.80530329	0.000174254

	10119 / FH) OX=722438 GN=dnaJ PE=4 SV=1		
A0A0H3DN85	ATP synthase subunit alpha OS=Mycoplasma pneumoniae (strain ATCC 15531 / DSM 22911 / NBRC 14401 / NCTC 10119 / FH) OX=722438 GN=atpA PE=3 SV=1	0.802230079	4.93281E-06
A0A0H3DKX2	50S ribosomal protein L6 OS=Mycoplasma pneumoniae (strain ATCC 15531 / DSM 22911 / NBRC 14401 / NCTC 10119 / FH) OX=722438 GN=rplF PE=3 SV=1	0.801024073	0.000113042
A0A0H3DNL1	Uncharacterized protein OS=Mycoplasma pneumoniae (strain ATCC 15531 / DSM 22911 / NBRC 14401 / NCTC 10119 / FH) OX=722438 GN=MPNE_0753 PE=4 SV=1	0.795075798	0.001951178
A0A0H3DMG5	Uridylate kinase OS=Mycoplasma pneumoniae (strain ATCC 15531 / DSM 22911 / NBRC 14401 / NCTC 10119 / FH) OX=722438 GN=pyrH PE=3 SV=1	0.794577805	0.007966106
P75227	Uncharacterized protein MG373 homolog OS=Mycoplasma pneumoniae (strain ATCC 29342 / M129) OX=272634 GN=MPN_551 PE=4 SV=1	0.794185471	6.27017E-05
A0A0H3DQ07	30S ribosomal protein S4 OS=Mycoplasma pneumoniae (strain ATCC 15531 / DSM 22911 / NBRC 14401 / NCTC 10119 / FH) OX=722438 GN=rpsD PE=3 SV=1	0.791254358	1.36125E-05
P75057	Spermidine/putrescine transport system permease protein PotC homolog OS=Mycoplasma pneumoniae (strain ATCC 29342 / M129) OX=272634 GN=potC PE=3 SV=1	0.790298289	0.000959452
A0A0H3DK29	Glutamyl-tRNA(Gln) amidotransferase, C subunit OS=Mycoplasma pneumoniae (strain ATCC 15531 / DSM 22911 / NBRC 14401 / NCTC 10119 / FH) OX=722438 GN=gatC PE=3 SV=1	0.79022358	0.005150449
A0A0H3DQ13	MG032/MG096/MG288 family 2 OS=Mycoplasma pneumoniae (strain ATCC 15531 / DSM 22911 / NBRC 14401 / NCTC 10119 / FH) OX=722438 GN=MPNE_0594 PE=4 SV=1	0.787541413	0.000149059
P75280	Uncharacterized lipoprotein MPN_506 OS=Mycoplasma pneumoniae (strain ATCC 29342 / M129) OX=272634 GN=MPN_506 PE=3 SV=1	0.787068907	1.65614E-05
A0A0H3DNJ7	Uncharacterized protein OS=Mycoplasma pneumoniae (strain ATCC 15531 / DSM 22911 / NBRC 14401 / NCTC 10119 / FH) OX=722438 GN=MPNE_0085 PE=4 SV=1	0.786772796	0.000202355
A0A0H3DP65	50S ribosomal protein L4 OS=Mycoplasma pneumoniae (strain ATCC 15531 / DSM 22911 / NBRC 14401 / NCTC 10119 / FH) OX=722438 GN=rplD PE=3 SV=1	0.785213869	0.000116047
P78035	50S ribosomal protein L1 OS=Mycoplasma pneumoniae (strain ATCC 29342 / M129) OX=272634 GN=rplA	0.783110627	0.000185548

	PE=3 SV=1		
P75461	Ribonucleoside-diphosphate reductase subunit beta OS=Mycoplasma pneumoniae (strain ATCC 29342 / M129) OX=272634 GN=nrdF PE=3 SV=1	0.781789809	2.52559E-05
A0A0H3DP88	Thymidylate synthase OS=Mycoplasma pneumoniae (strain ATCC 15531 / DSM 22911 / NBRC 14401 / NCTC 10119 / FH) OX=722438 GN=thyA PE=3 SV=1	0.780780305	0.000295391
E1QC64	Proline-rich P65 protein OS=Mycoplasma pneumoniae (strain ATCC 15531 / DSM 22911 / NBRC 14401 / NCTC 10119 / FH) OX=722438 GN=p65 PE=4 SV=1	0.779019228	0.027537929
Q50310	50S ribosomal protein L29 OS=Mycoplasma pneumoniae (strain ATCC 29342 / M129) OX=272634 GN=rpmC PE=3 SV=1	0.776106604	0.004936615
A0A0H3DKJ1	50S ribosomal protein L28 OS=Mycoplasma pneumoniae (strain ATCC 15531 / DSM 22911 / NBRC 14401 / NCTC 10119 / FH) OX=722438 GN=rpmB PE=3 SV=1	0.775025756	0.000131273
A0A0H3DKM6	DNA topoisomerase 4 subunit B OS=Mycoplasma pneumoniae (strain ATCC 15531 / DSM 22911 / NBRC 14401 / NCTC 10119 / FH) OX=722438 GN=parE PE=3 SV=1	0.773319992	0.002085156
P75460	Protein NrdI OS=Mycoplasma pneumoniae (strain ATCC 29342 / M129) OX=272634 GN=nrdI PE=3 SV=1	0.772065347	2.63761E-05
P11311	Adhesin P1 OS=Mycoplasma pneumoniae (strain ATCC 29342 / M129) OX=272634 GN=mgpA PE=1 SV=2	0.770112422	2.46621E-05
P75157	Uncharacterized lipoprotein MG439 homolog 5 OS=Mycoplasma pneumoniae (strain ATCC 29342 / M129) OX=272634 GN=MPN_640 PE=3 SV=1	0.763659522	2.12201E-06
P75610	Uncharacterized lipoprotein MPN_083 OS=Mycoplasma pneumoniae (strain ATCC 29342 / M129) OX=272634 GN=MPN_083 PE=3 SV=1	0.761345974	0.000127199
A0A0H3DN30	Uncharacterized protein OS=Mycoplasma pneumoniae (strain ATCC 15531 / DSM 22911 / NBRC 14401 / NCTC 10119 / FH) OX=722438 GN=MPNE_0517 PE=4 SV=1	0.758474362	8.64546E-06
A0A0H3DPB0	Uncharacterized protein OS=Mycoplasma pneumoniae (strain ATCC 15531 / DSM 22911 / NBRC 14401 / NCTC 10119 / FH) OX=722438 GN=MPNE_0288 PE=4 SV=1	0.757581139	0.001065904
P75542	Single-stranded DNA-binding protein OS=Mycoplasma pneumoniae (strain ATCC 29342 / M129) OX=272634 GN=ssb PE=3 SV=1	0.751505395	0.000779336
P75562	Uncharacterized protein MPN_107 OS=Mycoplasma pneumoniae (strain ATCC 29342 / M129) OX=272634	0.749013266	5.75543E-07

	GN=MPN_107 PE=4 SV=1		
P75378	Acyl carrier protein homolog OS=Mycoplasma pneumoniae (strain ATCC 29342 / M129) OX=272634 GN=MPN_406 PE=3 SV=1	0.74420917	4.31691E-05
A0A0H3DN64	50S ribosomal protein L14 OS=Mycoplasma pneumoniae (strain ATCC 15531 / DSM 22911 / NBRC 14401 / NCTC 10119 / FH) OX=722438 GN=rplN PE=3 SV=1	0.740969947	2.25061E-05
A0A0H3DNP5	Putative adhesin P1 OS=Mycoplasma pneumoniae (strain ATCC 15531 / DSM 22911 / NBRC 14401 / NCTC 10119 / FH) OX=722438 GN=MPNE_0237 PE=4 SV=1	0.737664108	0.004569322
A0A0H3DLG9	50S ribosomal protein L16 OS=Mycoplasma pneumoniae (strain ATCC 15531 / DSM 22911 / NBRC 14401 / NCTC 10119 / FH) OX=722438 GN=rplP PE=3 SV=1	0.733280144	2.93971E-05
A0A0H3DJY8	50S ribosomal protein L22 OS=Mycoplasma pneumoniae (strain ATCC 15531 / DSM 22911 / NBRC 14401 / NCTC 10119 / FH) OX=722438 GN=rplV PE=3 SV=1	0.732120664	0.000341436
P75289	Probable L-ribulose-5-phosphate 4-epimerase UlaF OS=Mycoplasma pneumoniae (strain ATCC 29342 / M129) OX=272634 GN=ulaF PE=3 SV=1	0.729336884	0.000688637
A0A0H3DMC3	Uncharacterized protein OS=Mycoplasma pneumoniae (strain ATCC 15531 / DSM 22911 / NBRC 14401 / NCTC 10119 / FH) OX=722438 GN=MPNE_0246 PE=4 SV=1	0.726477015	3.37867E-05
Q59550	ATP synthase subunit c OS=Mycoplasma pneumoniae (strain ATCC 29342 / M129) OX=272634 GN=atpE PE=3 SV=1	0.714927912	0.00935769
P75178	50S ribosomal protein L13 OS=Mycoplasma pneumoniae (strain ATCC 29342 / M129) OX=272634 GN=rplM PE=3 SV=1	0.711853251	6.4097E-05
P75237	30S ribosomal protein S20 OS=Mycoplasma pneumoniae (strain ATCC 29342 / M129) OX=272634 GN=rpsT PE=3 SV=1	0.711705887	0.004073364
A0A0H3DKV4	tRNA pseudouridine synthase A OS=Mycoplasma pneumoniae (strain ATCC 15531 / DSM 22911 / NBRC 14401 / NCTC 10119 / FH) OX=722438 GN=truA PE=3 SV=1	0.709411984	0.000946853
A0A0H3DLP6	50S ribosomal protein L3 OS=Mycoplasma pneumoniae (strain ATCC 15531 / DSM 22911 / NBRC 14401 / NCTC 10119 / FH) OX=722438 GN=rplC PE=3 SV=1	0.692867894	2.21924E-05
A0A0H3DPM4	Uncharacterized protein OS=Mycoplasma pneumoniae (strain ATCC 15531 / DSM 22911 / NBRC 14401 /	0.692128965	0.001415664

	NCTC 10119 / FH) OX=722438 GN=MPNE_0034 PE=4 SV=1		
Q50306	50S ribosomal protein L5 OS=Mycoplasma pneumoniae (strain ATCC 29342 / M129) OX=272634 GN=rplE PE=3 SV=1	0.687449419	8.25687E-05
A0A0H3DM13	Lon protease OS=Mycoplasma pneumoniae (strain ATCC 15531 / DSM 22911 / NBRC 14401 / NCTC 10119 / FH) OX=722438 GN=lon PE=2 SV=1	0.677491899	2.62036E-05
Q50307	50S ribosomal protein L24 OS=Mycoplasma pneumoniae (strain ATCC 29342 / M129) OX=272634 GN=rplX PE=3 SV=1	0.666815602	0.000332203
A0A0H3DLM1	Uncharacterized protein OS=Mycoplasma pneumoniae (strain ATCC 15531 / DSM 22911 / NBRC 14401 / NCTC 10119 / FH) OX=722438 GN=MPNE_0771 PE=4 SV=1	0.666386773	0.000204207
A0A0H3DLC2	30S ribosomal protein S7 OS=Mycoplasma pneumoniae (strain ATCC 15531 / DSM 22911 / NBRC 14401 / NCTC 10119 / FH) OX=722438 GN=rpsG PE=3 SV=1	0.657482549	0.002745988
P78020	50S ribosomal protein L31 OS=Mycoplasma pneumoniae (strain ATCC 29342 / M129) OX=272634 GN=rpmE PE=3 SV=1	0.646491396	0.034590292
Q59547	50S ribosomal protein L17 OS=Mycoplasma pneumoniae (strain ATCC 29342 / M129) OX=272634 GN=rplQ PE=3 SV=1	0.639955091	1.35456E-05
A0A0H3DKK7	DUF31 domain-containing protein OS=Mycoplasma pneumoniae (strain ATCC 15531 / DSM 22911 / NBRC 14401 / NCTC 10119 / FH) OX=722438 GN=MPNE_0095 PE=4 SV=1	0.634710889	8.75676E-07
P75458	50S ribosomal protein L27 OS=Mycoplasma pneumoniae (strain ATCC 29342 / M129) OX=272634 GN=rpmA PE=3 SV=1	0.63190915	0.00012063
A0A0H3DLK4	Mannitol-1-phosphate 5-dehydrogenase OS=Mycoplasma pneumoniae (strain ATCC 15531 / DSM 22911 / NBRC 14401 / NCTC 10119 / FH) OX=722438 GN=mtlD PE=3 SV=1	0.627452363	0.003337681
A0A0H3DKV6	Uncharacterized protein OS=Mycoplasma pneumoniae (strain ATCC 15531 / DSM 22911 / NBRC 14401 / NCTC 10119 / FH) OX=722438 GN=MPNE_0544 PE=4 SV=1	0.593947871	1.61775E-05
P75059	Spermidine/putrescine import ATP-binding protein PotA OS=Mycoplasma pneumoniae (strain ATCC 29342 / M129) OX=272634 GN=potA PE=3 SV=1	0.571207043	5.93079E-06
Q50341	Mgp-operon protein 3 OS=Mycoplasma pneumoniae (strain ATCC 29342 / M129) OX=272634 GN=MPN_142	0.520219432	1.48546E-06

	PE=1 SV=1		
P75156	Uncharacterized lipoprotein MG440 homolog 3 OS=Mycoplasma pneumoniae (strain ATCC 29342 / M129) OX=272634 GN=MPN_641 PE=3 SV=1	0.511040632	7.33335E-06
P75056	Uncharacterized lipoprotein MG045 homolog OS=Mycoplasma pneumoniae (strain ATCC 29342 / M129) OX=272634 GN=MPN_058 PE=3 SV=1	0.455175126	1.25501E-08
P75239	50S ribosomal protein L7/L12 OS=Mycoplasma pneumoniae (strain ATCC 29342 / M129) OX=272634 GN=rplL PE=3 SV=1	0.408093089	2.00983E-06
P75612	Putative ABC transporter ATP-binding protein MG065 homolog OS=Mycoplasma pneumoniae (strain ATCC 29342 / M129) OX=272634 GN=MPN_081 PE=3 SV=1	0.386273513	7.64866E-07
A0A0H3DN46	Uncharacterized protein OS=Mycoplasma pneumoniae (strain ATCC 15531 / DSM 22911 / NBRC 14401 / NCTC 10119 / FH) OX=722438 GN=MPNE_0751 PE=4 SV=1	0.367869794	2.13937E-07
A0A0H3DNA2	Efflux ABC transporter, permease protein OS=Mycoplasma pneumoniae (strain ATCC 15531 / DSM 22911 / NBRC 14401 / NCTC 10119 / FH) OX=722438 GN=MPNE_0091 PE=4 SV=1	0.322914237	1.99077E-06
A0A0H3DL10	Uncharacterized protein OS=Mycoplasma pneumoniae (strain ATCC 15531 / DSM 22911 / NBRC 14401 / NCTC 10119 / FH) OX=722438 GN=MPNE_0012 PE=4 SV=1	0.14234827	2.66654E-05
P75100	UPF0134 protein MPN_013 OS=Mycoplasma pneumoniae (strain ATCC 29342 / M129) OX=272634 GN=MPN_013 PE=3 SV=1	0.132658638	5.67489E-07
