# Supplementary material for: Quantitative proteomics analysis of Mycoplasma pneumoniae identifies potential macrolide resistance determinants
Source: AMB Express. 2021 Feb 12;11:26. doi: 10.1186/s13568-021-01187-8 (PMC7881084; doi:10.1186/s13568-021-01187-8)
Supplement: Supplementary file 3 — Additional file 3: Table S2. All KEGG annotation result of DEPs between macrolide-resistantstrain (C267) and macrolide-sensitivestrain (M129). [file 13568_2021_1187_MOESM3_ESM.docx]

**Table S2. All KEGG annotation result of DEPs between macrolide-resistantstrain (C267) and macrolide-sensitivestrain (M129)**

| Pathway Name | Count | Genes |
| --- | --- | --- |
| Ribosome | 28 | rpsZ、rplW、rpsB、rpsC、rplL、rpsH、rplO、rplU、rplJ、rplK、rplF、rpsD、rplD、rplA、rpmC、rpmB、rplN、rplP、rplV、rplM、rpsT、rplC、rplE、rplX、rpsG、rpmE、rplQ、rpmA |
| Metabolic pathways | 22 | deoD、dnaX、plsY、glpK、tmk、pdhC、csd、ulaE、atpF、nrdE、atpG、MPN_450、rpoE、atpA、pyrH、nrdF、thyA、thiI、ulaF、tpiA、atpE、nadK |
| Pyrimidine metabolism | 9 | deoD、dnaX、tmk、nrdE、MPN_450、rpoE、pyrH、nrdF、thyA |
| Aminoacyl-tRNA biosynthesis | 8 | hisS、valS、trpS、glyQS、leuS、pheT、metG、alaS |
| ABC transporters | 7 | ecfA2、ecfA1、MPN_611、pstA、potC、potA、MPN_058 |
| Purine metabolism | 6 | deoD、dnaX、nrdE、MPN_450、rpoE、nrdF |
| Biosynthesis of secondary metabolites | 5 | deoD、plsY、MPN_051、pdhC、tpiA |
| Oxidative phosphorylation | 4 | atpF、atpG、atpA、atpE |
| Homologous recombination | 4 | dnaX、MPN_450、ssb、ruvB |
| DNA replication | 4 | dnaX、dnaB、MPN_450、ssb |
| Microbial metabolism in diverse environments | 4 | pdhC、ulaE、ulaF、tpiA |
| Mismatch repair | 3 | dnaX、MPN_450、ssb |
| Protein export | 3 | secG、lspA、ftsY |
| Selenocompound metabolism | 2 | csd、metG |
| Glycerolipid metabolism | 2 | plsY、glpK |
| Nicotinate and nicotinamide metabolism | 2 | deoD、nadK |
| Ascorbate and aldarate metabolism | 2 | ulaE、ulaF、 |
| Glycerophospholipid metabolism | 2 | plsY、MPN_051 |
| Bacterial secretion system | 2 | secG、ftsY |
| Fructose and mannose metabolism | 2 | tpiA、mtlD |
| Glycolysis / Gluconeogenesis | 2 | pdhC、tpiA |
| Carbon metabolism | 2 | pdhC、tpiA |
| Biosynthesis of antibiotics | 2 | pdhC、tpiA |
| Two-component system | 1 | MPN_611 |
| Sulfur relay system | 1 | thiI |
| RNA polymerase | 1 | rpoE |
| Citrate cycle (TCA cycle) | 1 | pdhC |
| Base excision repair | 1 | mutM |
| One carbon pool by folate | 1 | thyA |
| RNA degradation | 1 | rnj |
| Pyruvate metabolism | 1 | pdhC |
| Biosynthesis of amino acids | 1 | tpiA |
